# Association of serum angiopoietin-like protein 2 with carotid intima-media thickness in subjects with type 2 diabetes

**DOI:** 10.1186/s12933-015-0198-z

**Published:** 2015-04-15

**Authors:** Chang Hee Jung, Woo Je Lee, Min Jung Lee, Yu Mi Kang, Jung Eun Jang, Jaechan Leem, Yoo La Lee, So Mi Seol, Hae Kyeong Yoon, Joong-Yeol Park

**Affiliations:** Asan Diabetes Center, University of Ulsan College of Medicine, Seoul, Republic of Korea; Asan Institute of Life Sciences, University of Ulsan College of Medicine, Seoul, Republic of Korea; Department of Internal Medicine, Asan Medical Center, University of Ulsan College of Medicine, Poongnap-dong, Songpa-gu, Seoul, 138-736 Republic of Korea

**Keywords:** Angiopoietin-like protein 2, Atherosclerosis, Carotid, Intima media thickness, Type 2 diabetes

## Abstract

**Background:**

Although recent animal studies have suggested that angiopoietin-like protein 2 (ANGPTL2), a novel inflammatory adipokine, is likely to be involved in the pathogenesis of atherosclerosis, in rodents, little is known regarding whether serum ANGPTL2 level is also associated with atherosclerosis in humans, especially in patients with type 2 diabetes. The aim of this study was to investigate whether serum ANGPTL2 concentration is associated with atherosclerosis by measuring carotid intima-media thickness (IMT) in subjects with type 2 diabetes without previous history of cardiovascular diseases. In addition, we examined the clinical and biochemical variables associated with serum ANGPLT2 concentration.

**Methods:**

We measured the circulating ANGPTL2 level in 166 subjects (92 men and 74 women; mean age of 60.0 years) with type 2 diabetes. Measurements of carotid IMT were performed in all subjects.

**Results:**

Serum ANGPTL2 concentration was positively correlated with carotid IMT (*r* = 0.220, *p* = 0.004). In multiple linear regression, serum ANGPTL2 concentration was independently associated with increased carotid IMT along with older age, male gender, and higher systolic blood pressure. Higher levels of hemoglobin A1c and high-sensitivity C-reactive protein were significantly associated with elevated serum ANGPTL2 concentration in subjects with type 2 diabetes.

**Conclusions:**

Serum ANGPTL2 concentration was significantly and positively associated with carotid atherosclerosis in patients with type 2 diabetes, suggesting that ANGPTL2 may be important in the atherosclerosis in humans.

**Electronic supplementary material:**

The online version of this article (doi:10.1186/s12933-015-0198-z) contains supplementary material, which is available to authorized users.

## Background

Obesity is now clearly associated with a state of chronic low-grade inflammation characterized by abnormal cytokine production and activation of inflammatory signaling pathways in adipose tissues, which contributes to insulin resistance [1]. Angiopoietin is a part of a family of vascular growth factors that are important modulators of angiogenesis [2]. A family of proteins structurally similar to angiopoietin was identified and designated as ‘angiopoietin-like proteins (ANGPTLs)’ [3-7]. Encoded by eight genes, ANGPTL1-8 are structurally similar to angiopoietins, characterized by a coiled-coil domain in the N-terminus and a fibrinogen-like domain in the C-terminus, except for ANGPTL8 which does not have a fibrinogen-like domain [3-7]. However, ANGPTLs do not bind to angiopoietin receptors, (i.e., either Tie2 or the related protein Tie1), suggesting that these ligands function differently from angiopoietins [6,8,9]. Several studies have reported that a subset of ANGPTLs function in glucose, lipid, and energy metabolism, although they mainly regulate angiogenesis [3,7].

Among ANGPTLs, ANGPTL2 is abundantly expressed in visceral adipose tissue and has been demonstrated to be involved in adipose tissue remodeling, such as angiogenesis and extracellular matrix remodeling, ultimately leading to adipogenesis and adipocyte hypertrophy, increasing excess energy storage in adipose tissue [4,10]. In cases of severe obesity, ANGPTL2 expression is abundant in adipose tissues and excess signaling by ANGPTLs leads to chronic inflammation, resulting in metabolic diseases such as obesity-related insulin resistance and type 2 diabetes [4,10]. Besides its metabolic role, ANGPTL2 has growingly received attention due to its deleterious effect on atherosclerotic diseases [9-14]. In a mouse model, ANGPTL2-deficient animals showed decreased abdominal aortic aneurysms, characterized pathologically by atherosclerotic changes accompanying chronic inflammation and infiltrating inflammatory cells [13]. In addition, perivascular ANGPTL2 accelerates neointimal hyperplasia after endovascular injury in mice [12]. Recently, disruption of ANGPTL2 in apolipoprotein E-deficient mice (*ApoE*^*-/-*^*/Angptl2*^*-/-*^) was found to attenuate atherosclerosis progression by decreasing the number of macrophage infiltrating atheromatous plaques, thereby reducing vascular inflammation [9]. Although these findings support the concept that ANGPTL2 is more likely to be involved in the regulation of vascular function, more specifically in atherosclerosis in rodents [9,10,12-14], few studies have examined the relevance of circulating ANGPTL2 levels to subclinical atherosclerosis in humans [9]. Furthermore, it remains unclear about to what extent ANGPTL2 contributes to the development of atherosclerosis independently of conventional cardiovascular risk factors, especially in subjects with type 2 diabetes.

In our current study, we investigated whether serum ANGPTL2 concentration was associated with the parameters of atherosclerosis in subjects with type 2 diabetes using carotid artery intima-media thickness (IMT), a non-invasive index of early atherosclerosis [15]. In addition, we tried to identify the clinical and biochemical variables related to the serum concentration of ANGPTL2.

## Methods

### Study populations

As previously described [16], we consecutively recruited patients who visited the Asan Diabetes Center at Asan Medical Center (AMC), Seoul, Republic of Korea between November 2012 and January 2014 for treatment of diabetes and evaluation of micro- and macrovascular complications of diabetes. Before starting the recruitment process, the study was registered at the Clinical Research Information Service (cris.nih.go.kr) (KCT0000598). Our study population comprised 203 subjects who had undergone carotid artery ultrasonography during recruitment.

A single specially trained nurse interviewed all participants and obtained information on medications and history of previous medical or surgical diseases. A history of cardiovascular disease (CVD) was defined as a history of physician-diagnosed CVD (eg, previous myocardial infarction, angina, coronary-artery bypass grafting, and/or stroke). After exclusion of subjects with a history of CVD (n = 37), a total of 166 subjects (92 men aged 38-78 years [mean age, 59.7 years] and 74 women aged 38-76 years [mean age, 60.4 years]) with type 2 diabetes were included in the study. All subjects provided written informed consent and the study was approved by the Institutional Review Board of the AMC.

Antidiabetic treatments were categorized as none, oral hypoglycemic agents (OHAs), or insulin with or without OHAs. Peroxisome proliferator-activated receptor γ agonists such as thiazolidinedione affect the expression of ANGPTL2 [10], therefore patients treated with thiazolidinedione were excluded from the study prior to the initiation of the recruitment process. Antihypertensive medications included any of the following: angiotensin-converting enzyme inhibitors, angiotensin II receptor blockers, β-blockers, calcium-channel blockers, diuretics, or α-blockers.

### Anthropometric and laboratory measurements

Height and weight were obtained while subjects wore light clothing without shoes. The waist circumference (WC, cm) was measured midway between the costal margin and the iliac crest at the end of a normal expiration. The blood pressure (BP) was measured on the right arm after a rest of ≥5 min.

After overnight fasting, early morning blood samples were drawn from the antecubital vein into vacuum tubes and subsequently analyzed by a central, certified laboratory at the AMC. Measurements included fasting plasma glucose (FPG), hemoglobin A1c (HbA1c), insulin, high-sensitivity C-reactive protein (hsCRP), several lipid parameters, and liver enzyme levels.

FPG was measured by the glucose oxidase method using a Toshiba 200FR Neo (Toshiba Medical Systems Co., Ltd., Tokyo, Japan). HbA1c was measured by high-performance liquid chromatography (HPLC) using a Variant II Turbo (Bio-Rad Laboratories, Hercules, CA). Fasting total cholesterol, high-density lipoprotein-cholesterol (HDL-C), low-density lipoprotein-cholesterol (LDL-C), triglyceride (TG), uric acid, aspartate aminotransferase (AST), and alanine aminotransferase (ALT) were measured using an enzymatic colorimetric method (Toshiba Medical Systems). HsCRP was measured using the immunoturbidimetric method (Toshiba Medical Systems). Serum insulin was measured by immunoradiometric assay (TFB Co., Ltd, Tokyo, Japan). Homeostatic model assessment of insulin resistance (HOMA-IR) was used as a surrogate measure of systemic insulin resistance, which has been suggested to be a useful test for the evaluation of insulin resistance even in patients with type 2 diabetes treated with an insulin secretagogue or insulin [17,18]. All enzyme activities were measured at 37°C. HOMA-IR was calculated according to the following equation: HOMA-IR = [Insulin (μU/mL) × FPG (mmol/L)]/22.5.

The extent of albuminuria was determined from urinary albumin-to-creatinine ratio (UACR), which was measured by a photometric method using the Integra 800 system (Roche Diagnostics, Indianapolis, IN) in a random spot urine collection. Creatinine was measured using the Jaffe method, and estimated glomerular filtration rate (eGFR) was calculated using the modified Modification of Diet in Renal Disease (MDRD) equation [19].

### Measurement of serum concentrations of ANGPTL 2 and total adiponectin

Fasting venous blood samples were centrifuged, and the supernatants were carefully collected to exclude cell components. All samples with hemolysis or clotting were discarded. Serum samples were stored at −80°C until use. Serum ANGPTL2 concentrations were measured using a human ANGPTL2 sandwich enzyme-linked immunosorbent assay (ELISA) kit (Immuno-Biological Laboratories Co., Ltd, Japan) with two mouse monoclonal antibodies that were confirmed to recognize only ANGPTL2 and not react with any other ANGPTLs or angiopoietins [10]. The kit’s lower detection limit is typically less than 0.01 ng/ml. The intra-and inter-assay coefficients of variations (CVs) of the ELISA kit were <5.9% and <10.5%, respectively. Serum total adiponectin concentration was also measured using commercially available ELISA kits (Adipogen, Seoul, South Korea), with intra-and inter-assay CVs of <4.0% and <6.0%, respectively. Samples were assayed in duplicate and all results were reported as means.

### Ultrasonographic measurement of carotid IMT

Ultrasound analysis was performed by a single specialized technician who was blind to the patient's clinical data. The examination was carried out according to a previously established protocol standardized for both carotid arteries [20]. Images were acquired at end diastole (defined as the R wave of the electrocardiogram) using a 5-12 MHz linear transducer and high resolution ultra-sound (HD 11 XE, Philips Healthcare, Andover MA). All measurements were recorded with the subject in a supine position with the head elevated to 45° and tilted to either side by 30°. The IMT was measured over a 1-cm segment of the artery located approximately 0.5 cm below the carotid-artery bulb and considered not to contain any plaque (i.e., not to have any perceivable protrusion of the artery wall into the lumen) [21,22]. For the purpose of the present study, a QLAB IMT-quantification software measurement plug-in (Philips Healthcare) was used to increase the consistency and reliability of IMT measurements, reduce the effort required to successfully carry out IMT measurements, and minimize the time needed to complete an IMT study [23]. The correlation coefficient for carotid IMT was 0.985. To quantify carotid artery wall thickness we used the mean of the right and left IMTs in the present study.

### Statistical analysis

Continuous variables with normal distribution were expressed as mean ± SD, whereas continuous variables with skewed distribution were expressed as median (and interquartile range). Categorical variables were expressed as proportions (%). The serum ANGPTL2 tertiles were as follows: Q1 ≤ 3.27 ng/mL, Q2 = 3.28-5.19 ng/mL, and Q3 ≥ 5.20 ng/mL. Demographic and biochemical characteristics of the study population according to the serum ANGPTL2 tertiles were compared using a one-way analysis of variance (ANOVA) with the Scheffe’s method as a *post-hoc* analysis, or the Kruskal-Wallis test with the Dunn procedure for continuous variables, and the chi-squared test for categorical variables. The correlation between serum ANGPTL2 and various parameters, including carotid IMT, was evaluated by Pearson correlation analysis. Logarithmic (log) transformation of values showing a skewed distribution was carried out before the correlation analysis. The association between the mean carotid IMT and serum ANGPTL2 level was investigated using backward multiple linear regression, with mean carotid IMT serving as the dependent variable. To identify the independent determinants of carotid IMT, univariate regression analysis was performed as a preliminary step to a multiple linear regression. In the multivariate model, all the variables that were associated with the carotid IMT with *p* values < 0.20 in the univariate regression analysis were included. To identify the factors contributing to increased serum ANGPTL2 levels, a backward multiple linear regression was performed with serum ANGPTL2 levels serving as the dependent variable. This analysis was adjusted for variables, which were associated with serum ANGPTL2 with *p* values < 0.20 in the univariate regression analysis. All independent variables in the multiple linear regression analysis were tested for multicollinearity; if the variance inflation factor (VIF) exceeded 10, the variable was considered to be collinear. All statistical analyses were carried out with SPSS version 19.0 for Windows (IBM Corp., Armonk, NY). A two-tailed *p*-value < 0.05 was regarded as statistically significant

## Results

The average age of the study subjects was 60.0 ± 8.3 years, and about 60% of subjects were obese with a mean BMI of 26.0 ± 3.4 kg/m^2^ [24]. The mean concentration of serum ANGPTL2 was 4.75 ± 2.63 ng/ml. The clinical and biochemical characteristics of the study subjects according to the serum ANGPTL2 tertile are shown in Table 1. A number of variables, including age, sex, BMI, WC, systolic BP, diastolic BP, current smoker, duration of diabetes, antidiabetic medications, percentage of subjects taking antihypertensive medications or statins, levels of total cholesterol, LDL-C, uric acid, AST, ALT, hsCRP, UACR, eGFR, and total adiponectin, were similar among the three groups (Table 1). FPG, HbA1c, TG, HOMA-IR, and carotid IMT linearly increased (*p* value 0.047 to 0.001), while HDL-C decreased linearly across increasing serum ANGPTL2 tertile categories (*p* = 0.036).

**Table 1:** **Clinical biochemical characteristics of the study subjects according to the serum ANGPTL2 tertile**

	**Serum ANGPTL2 tertile**			
	**Q1**	**Q2**	**Q3**	***P*** **value** ^**a**^
**Variables**	**(≤3.27ng/ml)**	**(3.28 ~ 5.19 ng/ml)**	**(≤5.20 ng/ml)**	
N	55	56	55	–
Age (years)	60.3 **±** 7.1	60.8 **±** 8.3	58.9 **±** 9.4	0.429
Sex (male, %)	65.5	44.6	56.4	0.085
BMI (kg/m^2^)	25.3 **±** 2.7	26.5 **±** 3.6	26.2 **±** 3.7	0.155
WC (cm)	88.4 **±** 9.6	91.0 **±** 9.0	90.2 **±** 7.7	0.281
Systolic BP (mmHg)	126.9 **±** 17.0	126.8 **±** 15.5	128.4 **±** 14.1	0.823
Diastolic BP (mmHg)	75.2 **±** 9.1	74.6 **±** 8.4	76.2 **±** 8.5	0.625
Current smoker (%)	21.8	12.5	20.0	0.383
Duration of diabetes (years)	12 (4 − 14)	9 (6 − 14)	8 (4 − 13)	0.225
Antidiabetic medication (%)				0.400
None	7.3	1.8	7.3	
OHA	74.5	85.7	81.8	
Insulin ± OHA	18.2	12.5	10.9	
Antihypertensive medication (%)	52.7	66.1	56.4	0.336
Statin use (%)	76.4	71.4	76.4	0.790
FPG (mmol/L)	7.4 ± 2.0^b^	7.8 ± 1.8^b,c^	8.2 ± 2.0^c^	0.042
HbA1c (%)	6.8 (6.4 − 7.2)^b^	7.0 (6.3 − 7.9)^b,c^	7.3 (6.7 − 8.2)^c^	0.047
HbA1c (mmol/mol)	51 (46 − 55)^b^	53 (45 − 63)^b,c^	56 (50 − 66)^c^	0.047
Total cholesterol (mmol/L)	4.1 ± 1.0	4.0 ± 0.8	4.0 ± 0.8	0.615
TG (mmol/L)	1.1 (0.9 − 2.1)	1.4 (1.1 − 2.2)	1.6 (1.1 − 2.4)	0.037
LDL-C (mmol/L)	2.5 ± 0.7	2.5 ± 0.7	2.5 ± 0.7	0.897
HDL-C (mmol/L)	1.3 ± 0.3^b^	1.2 ± 0.3^b,c^	1.1 ± 0.3^c^	0.036
Uric acid (μmol/L)	297.4 **±** 89.2	285.5 **±** 71.4	297.4 **±** 71.4	0.781
AST (U/L)	21 (18 − 26)	23 (20 − 31)	23 (20 − 34)	0.079
ALT (U/L)	18 (14 − 25)	21 (16 − 38)	20 (15 − 33)	0.205
hsCRP (mg/L)	0.5 (0.1 − 1.1)	0.6 (0.3 − 1.1)	0.6 (0.4 − 1.6)	0.130
UACR (mg/g)	7.0 (4.0 − 24.8)	12.8 (5.7 − 23.9)	9.3 (4.9 − 26.7)	0.440
eGFR (ml/min/1.73m^2^)	91.8 **±** 22.5	94.6 **±** 22.1	91.8 **±** 20.6	0.726
HOMA-IR	2.2 (1.2 − 3.7)^b^	2.5 (1.6 − 4.5)^b,c^	2.9 (1.7 − 5.3)^c^	0.027
Adiponectin (μg/ml)	9.1 **±** 5.4	9.9 **±** 7.3	8.5 **±** 4.7	0.465
Mean IMT (mm)	0.66 **±** 0.12^b^	0.67 **±** 0.10^b^	0.73 **±** 0.12	0.001

^a^difference among three groups.

^b,c^the same letters indicate a statistically insignificant difference.

Table 2 shows the correlation between serum levels of ANGPTL2 versus various glucometabolic parameters. In the Pearson’s correlation analysis, the ANGPTL2 levels showed a positive correlation with AST (*r* = 0.222, *p* = 0.004), hsCRP (*r* = 0.218, *p =* 0.011), HbA1c (*r* = 0.178, *p* = 0.022), and HOMA-IR (*r* = 0.116, *p* = 0.045) following log transformation of all four factors.

**Table 2:** **Correlation between serum levels of ANGPTL2 and glucometabolic parameters**

	**ANGPTL2 (ng/ml)**	
**Variables**	***r***	***P*** **value**
BMI	0.093	0.231
WC	0.047	0.551
SBP	0.003	0.968
DBP	0.014	0.859
FPG	0.144	0.064
HbA1c^a^	0.178	0.022
Duration of diabetes	−0.136	0.080
Total cholesterol	−0.021	0.786
TG^a^	0.148	0.057
LDL-C	−0.005	0.949
HDL-C	−0.137	0.078
Uric acid	−0.026	0.740
AST^a^	0.222	0.004
ALT^a^	0.142	0.068
hsCRP^a^	0.218	0.011
UACR^a^	0.084	0.281
eGFR	−0.056	0.475
HOMA-IR^a^	0.116	0.045
Adiponectin	−0.096	0.235

^a^Logarithmic transformation was performed. NS: not significant.

**Figure 1:**
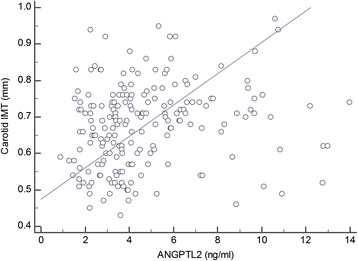
Correlation between the serum ANGPLT2 concentrations and carotid IMT (*r* = 0.220, *p* = 0.004).

In the Pearson’s correlation analysis, the ANGPTL2 level was positively correlated with carotid IMT (*r* = 0.220, *p* = 0.004, Figure 1). The correlation between serum ANGPTL2 level and carotid IMT was significant even after adjusting for age (*r* = 0.272, *p* < 0.001).

As shown in Table 3, multiple linear regression demonstrated that serum ANGPTL2 [*B* (SE) = 0.012 (0.003); *p* < 0.001] and other clinical parameters, including age [*B* (SE) = 0.005 (0.001); *p* < 0.001], male gender [(vs. female), *B* (SE) = 0.060 (0.015); *p* < 0.001], and systolic BP [*B* (SE) = 0.001 (0.001); *p =* 0.016], were independently associated with carotid IMT.

**Table 3:** **Multiple linear regression with the mean carotid IMT as a dependent variable**

		**Univariate analysis**			**Multivariate analysis**	
**Variables**	***β***	***B*** **(SE)**	***P*** **value**	***β***	***B*** **(SE)**	***P*** **value**
Age (years)	0.376	0.005 (0.001)	<0.001	0.374	0.005 (0.001)	<0.001
Male (vs. female)	0.235	0.054 (0.018)	0.002	0.257	0.060 (0.015)	<0.001
BMI	0.093	0.003 (0.003)	0.234			
WC	0.176	0.002 (0.001)	0.023			
Systolic BP	0.242	0.002 (0.001)	0.002	0.165	0.001 (0.001)	0.016
Diastolic BP	0.177	0.002 (0.001)	0.023			
Current smoker^a^	−0.009	−0.003 (0.023)	0.908			
Duration of diabetes (years)	0.146	0.003 (0.001)	0.060			
HTN medication use^b^	0.185	0.043 (0.018)	0.017			
Statin use^b^	−0.060	−0.016 (0.021)	0.443			
FPG	0.076	0.000 (0.000)	0.329			
HbA1c^c^	0.089	0.080 (0.070)	0.253			
Total cholesterol	−0.006	0.000 (0.000)	0.938			
TG^d^	−0.026	−0.006 (0.018)	0.737			
LDL-C	0.000	0.000 (0.000)	0.995			
HDL-C	−0.048	0.000 (0.001)	0.538			
Uric acid	0.162	0.014 (0.007)	0.037			
AST^c^	0.044	0.014 (0.024)	0.572			
ALT^c^	0.051	0.011 (0.016)	0.511			
hsCRP^c^	0.057	0.006 (0.009)	0.513			
UACR^c^	−0.004	0.000 (0.007)	0.958			
eGFR	−0.153	−0.001 (0.000)	0.049			
HOMA-IR^c^	−0.029	−0.003 (0.010)	0.730			
Adiponectin	−0.095	−0.002 (0.002)	0.224			
Serum ANGPTL2	0.220	0.010 (0.003)	0.004	0.263	0.012 (0.003)	<0.001

β: standardized coefficient, B: unstandardized coefficient, SE: standard error.

^a^vs. noncurrent smoker.

^b^vs. nonuser.

^c^Logarithmic transformation was performed.

Finally, we examined the clinical and biochemical factors that contributed to the elevated serum ANGPTL2 concentrations in our subjects with type 2 diabetes. Multiple linear regression demonstrated that higher HbA1c [*B* (SE) = 3.648 (1.789); *p =* 0.043] and hsCRP [*B* (SE) = 0.439 (0.196); *p* = 0.027] were significantly associated with elevated serum ANGPTL2 concentrations in these individuals (Additional file 1: Table S1).

## Discussion

ANGPTL2 is a novel proinflammatory cytokine primarily secreted by adipose tissues, although vascular endothelial cells and monocyte/macrophages also secrete this protein [9,10,13]. ANGPTL2 has been known to regulate angiogenesis similarly to several other ANGPTLs [3,4]. In addition, ANGPTL2 has the unique capacity to induce an inflammatory response in blood vessels as well as in adipose tissue [9,10,12-14]. For instance, several experimental studies have suggested the biological relevance of ANGPTL2 as a regulator of atherosclerosis in rodents [9,10,12-14]. In our current study, the serum ANGPTL2 concentration is significantly and positively associated with carotid IMT in subjects with type 2 diabetes (*r* = 0.220, *p* < 0.01, Figure 1). Furthermore, this association is independent of conventional cardiovascular risk factors (Table 3). Considering the reported harmful effects of ANGPTL2 on vascular inflammation in rodents [9,10,12-14], our current findings provide further evidence that this protein may play a key role in atherosclerosis in humans.

To date, there have been a few studies that have measured the serum ANGPTL2 concentrations in human subjects [9,10,14,25-27] and investigated the differences in these levels according to the presence of CVD [9,10,14]. In 2009, Tabata et al. measured the serum levels of ANGPTL2 in a total of 109 patients with coronary heart disease (CHD) diagnosed by coronary angiography and 89 drug-naïve type 2 diabetes patients [10]. These authors demonstrated that serum ANGPTL2 was significantly increased in patients with CHD or type 2 diabetes [10]. More recently, Horio et al. demonstrated that ANGPTL2 was abundantly expressed in atheromatous plaques in human coronary artery, and that the circulating ANGPTL2 levels measured by ELISA were higher in human subjects with CHD (defined by coronary artery stenosis ≥75% on coronary angiography; n = 89) compared with age-matched non-CHD subjects (n = 30) [9]. Furthermore, they showed that serum ANGPTL2 levels were associated with carotid IMT in 359 seniors aged between 85 to 99 years with no history of CVD, but with relatively reduced eGFR (mean 62.5 ± 16.8 ml/min/1.73m^2^) and advanced stages of atherosclerosis (mean carotid IMT of 0.99 ± 0.15 mm compared with our current study measurement of 0.69 ± 0.12 mm), among whom a small proportion of subjects with diabetes was included (approximately 18.4%) [9]. Although these previous data support the hypothesis that serum ANGPTL2 is strongly related to atherosclerosis in humans, little is known whether this role of ANGPTL2 applies in purely type 2 diabetes cases and/or in subject with early stage atherosclerosis. Our current evidence may suggest the independent involvement of ANGPTL2 in the pathogenesis of early subclinical atherosclerosis in humans, especially in subjects with type 2 diabetes. This role of ANGPTL2 in early atherosclerosis accords with the findings of a previous animal study, in which ANGPTL2 altered the release of nitric oxide, an important anti-atherogenic molecule [28], leading to endothelial dysfunction at stages of atherosclerosis preceding atherosclerotic plaque formation [9,14]

In humans, the ANGPTL2 concentration in the circulation is up-regulated in obesity (particularly visceral obesity) and is correlated with the levels of systemic insulin resistance [10,25]. In accordance with these previous results [10,25], biochemical variables indicating insulin-resistant profiles such as higher TG and HOMA-IR and lower HDL-C could be seen across increasing serum ANGPTL2 tertile categories in our present analyses (Table 1). In addition, we found a significant positive correlation between serum ANGPTL2 concentration and AST, hsCRP, and HOMA-IR (Table 2). Our current findings thus support the previously suggested possibility that circulating ANGPTL2 levels can serve as a marker of obesity-induced metabolic abnormalities as well as atherosclerosis [10,25,29].

There have been conflicting results regarding the role of ANGPTL2 in glucose metabolism [10,30]. In a previous study using ANGPTL2 knock-out mice, insulin sensitivity was improved in both the skeletal muscle and liver in mice fed with a high-fat diet [10]. In contrast, the replenishment of ANGPTL2 stimulated insulin sensitivity and improved glucose tolerance in mice [30]. In our current study, glycemic variables including FPG and HbA1c increased as serum ANGPTL2 tertile categories increased (Table 1), and there was a positive correlation observed between the serum ANGPTL2 concentration and HbA1c (Table 2), which are in line with the results of a previous study [25]. Furthermore, we observed that HbA1c is one of the independent contributors to the increased serum ANGPTL2 concentration (Additional file 1: Table S1). Recently, Doi et al. firstly demonstrated that elevated serum ANGPTL2 levels were positively associated with the development of type 2 diabetes in a total of 2,164 community-dwelling Japanese individuals, independent of other risk factors including hsCRP levels [31]. In that analysis, the risk of developing type 2 diabetes was significantly higher in the highest ANGPTL2 quartile (i.e., ≥ 3.41 ng/ml) than in the lowest quartile (hazard ratio, 1.80) [31]. Although further studies are needed to reveal the role of ANGPTL2 in the pathogenesis of type 2 diabetes, these earlier results and our present findings suggest the possibility that ANGPTL2 might be one of key mediators that link obesity and type 2 diabetes.

It is well accepted that obesity can be regarded as a state of chronic subclinical inflammation [32-34], and levels of hsCRP, a marker of low-grade inflammation, have been previously linked to visceral obesity [35,36]. The liver is assumed to be the major source of CRP production; however, adipose tissues have also been suggested as a direct source of CRP [37,38]. In our current study, the serum ANGPTL2 concentration showed a significant correlation with hsCRP (Table 2), and hsCRP was one of the independent contributors to the increased serum ANGPTL2 concentration (Additional file 1: Table S1), which are in accordance with the results of a previous study [10]. This close relationship between ANGPTL2 and hsCRP further implicates that ANGPTL2 is one of key adipocyte-derived inflammatory mediators linking obesity to related metabolic diseases.

This study has several noteworthy limitations. First, our sample size was not large enough to make any definite conclusions. This limitation was partly reflected in the results of our study, in which there was an insignificant inverse correlation between the levels of total serum adiponectin and ANGPTL2 (*r* = −0.096, *p* = 0.235 in Table 2), which is in contrast to the results of a previous study showing a significant association between these two factors [39]. Second, we lacked data on other inflammatory markers of systemic inflammation besides hsCRP. Additional markers reflecting adipose tissue dysfunction such as IL-6 would significantly strengthen the findings of this study [40]. Third, due to the cross-sectional design of our present study, we could not assess the temporal relationship between the serum ANGPTL2 level and increased carotid atherosclerosis. Fourth, because we included only Korean subjects, our results may not be applicable to other ethnic populations.

Despite these limitations, our current study is meaningful in that we demonstrated the relationships between serum ANGPTL2 concentration and early subclinical atherosclerosis in humans, especially in subjects with type 2 diabetes.

## Conclusions

In conclusion, we found that the serum ANGPTL2 concentration is significantly and positively associated with carotid IMT in human subjects with type 2 diabetes. This further supports the recently proposed notion that ANGPTL2 may be an important factor in the pathogenesis of atherosclerosis in humans [9,10,14], similarly to its possible role in the regulation of vascular function in rodents [9,10,12-14]. Further prospective studies are needed to determine whether ANGPTL2 is a useful predictive marker for CVD.

## Additional file

Additional file 1: Table S1. Univariate and multivariate analysis of the relationship between the serum ANGPTL2 concentrations and various clinical parameters.

## Abbreviations

ALT: Alanine aminotransferase
AMC: Asan medical center
ANGPTL: Angiopoietin-like protein
ANOVA: One-way analysis of variance
AST: Aspartate aminotransferase
BP: Blood pressure
CHD: Coronary heart disease
CV: Coefficients of variation
CVD: Cardiovascular disease
eGFR: Estimated glomerular filtration rate
ELISA: Enzyme-linked immunosorbent assay
FPG: Fasting plasma glucose
HbA1c: Hemoglobin A1c
HDL-C: High-density lipoprotein-cholesterol
HOMA-IR: Homeostatic model assessment of insulin resistance
HPLC: High-performance liquid chromatography
hsCRP: High-sensitivity C-reactive protein
IMT: Intima-media thickness
LDL-C: Low-density lipoprotein-cholesterol
MDRD: Modified modification of diet in renal disease
OHAs: Oral hypoglycemic agents
TG: Triglyceride
UACR: Urinary albumin-to-creatinine ratio
VIF: Variance inflation factor
WC: Waist circumference
