# Supplementary material for: Association of serum angiopoietin-like protein 2 with carotid intima-media thickness in subjects with type 2 diabetes
Source: Cardiovasc Diabetol. 2015 Apr 15;14:35. doi: 10.1186/s12933-015-0198-z (PMC4404615; doi:10.1186/s12933-015-0198-z)
Supplement: Additional file 1: Table S1. — Univariate and multivariate analysis of the relationship between the serum ANGPTL2 concentrations and various clinical parameters. [file 12933_2015_198_MOESM1_ESM.docx]

**Additional file 1: Table S1.** Univariate and multivariate analysis of the relationship between the serum ANGPTL2 concentrations and various clinical parameters

|  |  | **Univariate analysis** | |  | **Multivariate analysis** | |
| --- | --- | --- | --- | --- | --- | --- |
| **Variables** | ***β*** | ***B* (SE)** | ***P* value** | ***β*** | ***B* (SE)** | ***P* value** |
| Age (years) | −0.084 | −0.027 (0.025) | 0.280 |  |  |  |
| Male (vs. female) | −0.035 | −0.183 (0.412) | 0.658 |  |  |  |
| BMI | 0.093 | 0.073 (0.061) | 0.231 |  |  |  |
| WC | 0.047 | 0.014 (0.023) | 0.551 |  |  |  |
| Systolic BP | 0.003 | 0.001 (0.013) | 0.968 |  |  |  |
| Diastolic BP | 0.014 | 0.004 (0.024) | 0.859 |  |  |  |
| Current smoker^a^ | 0.039 | 0.268 (0.532) | 0.615 |  |  |  |
| Duration of diabetes (years) | −0.136 | −0.054 (0.031) | 0.080 |  |  |  |
| HTN medication use^b^ | 0.027 | 0.142 (0.416) | 0.732 |  |  |  |
| Statin use^b^ | −0.008 | −0.049 (0.471) | 0.917 |  |  |  |
| FPG | 0.144 | 0.011 (0.006) | 0.064 |  |  |  |
| HbA1c^c^ | 0.178 | 3.641 (1.575) | 0.022 | 0.172 | 3.648 (1.789) | 0.043 |
| Total cholesterol | −0.021 | −0.002 (0.006) | 0.786 |  |  |  |
| TG^c^ | 0.148 | 0.780 (0.407) | 0.057 |  |  |  |
| LDL-C | −0.005 | 0.000 (0.007) | 0.949 |  |  |  |
| HDL-C | −0.137 | −0.031 (0.017) | 0.078 |  |  |  |
| Uric acid | −0.026 | −0.052 (0.156) | 0.740 |  |  |  |
| AST^c^ | 0.222 | 1.586 (0.545) | 0.004 |  |  |  |
| ALT^c^ | 0.142 | 0.679 (0.369) | 0.068 |  |  |  |
| hsCRP^c^ | 0.218 | 0.515 (0.199) | 0.011 | 0.186 | 0.439 (0.196) | 0.027 |
| UACR^c^ | 0.084 | 0.165 (0.152) | 0.281 |  |  |  |
| eGFR | −0.056 | −0.007 (0.009) | 0.475 |  |  |  |
| HOMA-IR^c^ | 0.116 | 0.323 (0.231) | 0.045 |  |  |  |
| Adiponectin | −0.093 | −0.041 (0.035) | 0.235 |  |  |  |

β: standardized coefficient, B: unstandardized coefficient, SE: standard error

^a^vs. noncurrent smoker

^b^vs. nonuser

^c^Logarithmic transformation was performed.
